# 2012年美国临床肿瘤学会年会——小细胞肺癌治疗研究进展

**DOI:** 10.3779/j.issn.1009-3419.2012.11.11

**Published:** 2012-11-20

**Authors:** 侃 吴

**Affiliations:** 1 310053 杭州，浙江中医药大学第二临床医学院 The Second Clinical Medical College, Zhejiang Chinese Medical University, Hangzhou 310053, China; 2 310006 杭州，杭州市第一人民医院医疗集团，杭州市肿瘤医院放疗科 Department of Radiation Oncology, Hangzhou First People's Hospital, Hangzhou Cancer Hospital, Hangzhou 310006, China

第48届美国临床肿瘤学会（American Society of Clinical Oncology, ASCO）年会于2012年6月1日-5日在美国芝加哥召开，现就本次大会关于小细胞肺癌（small cell lung cancer, SCLC）治疗的研究进展作一介绍。

## 1 SCLC的一线治疗

### 1.1 局限期SCLC的治疗

化疗（4个周期EP方案：依托泊苷+顺铂）联合胸部超分割放疗是目前局限期SCLC的标准治疗方案。2002年日本的JCOG9511研究^[1]^显示在广泛期SCLC治疗中，IP方案（伊立替康+顺铂）的总有效率和总生存（overall survival, OS）均优于EP方案。因此，日本的JCOG0202研究^[2]^尝试将IP方案用于局限期SCLC，该试验入组的281例患者接受了1个周期EP方案化疗（依托泊苷100 mg/m^2^ d1-d3 +顺铂80 mg/m^2^ d1）和同步胸部超分割放疗（45 Gy/30 f/15 d/3 w），治疗后获得缓解或疾病稳定的患者被随机分入两组，分别接受3个周期EP或者IP方案（伊立替康60 mg/m^2^+顺铂60 mg/m^2^ d1, d8, d15）巩固化疗。主要终点指标是OS。结果显示IP方案未能使患者获得OS的获益（危险比IP *vs* EP：1.085，95%CI：0.80-1.46），因此4个周期EP方案化疗同步加速超分割胸部放疗仍然是局限期SCLC标准治疗方案。

2012年版SCLC的美国国立综合癌症网络（National Comprehensive Cancer Network, NCCN）指南中推荐胸部放疗尽早开始，一般在前2个化疗周期开始，但一项来自韩国的研究对这一观点提出了挑战。韩国首尔三星医学中心的Park等^[3]^对于在化疗不同周期联合胸部放疗进行了研究。入组的219例局限期SCLC患者被随机分入两组，第1组在化疗的第1周期联合胸部放疗，第2组在化疗第3周期联合胸部放疗。化疗方案是4个周期的EP方案（顺铂70 mg/m^2^ d1+依托泊苷100 mg/m^2^ d1-d3，每3周为1个周期），胸部放疗计划为52.5 Gy/25 f/25 d/5 w。结果显示对于局限期SCLC患者的同步放化疗，放疗可以推迟到第3周期EP方案化疗开始时进行，推迟放疗不仅未降低中位OS（24.1个月*vs* 26.8个月，*P*=0.60）和完全缓解率（36% *vs* 38%, *P*=0.77），而且减少了严重不良事件的发生率，如发热性中性粒细胞减少症明显减少（21.6% *vs* 10.2%, *P*=0.02）。研究者认为放疗从第1周期化疗开始在实际应用中常常不能顺利进行，有时强制放疗与化疗同时开始反而会造成总治疗时间的延长；而且，对于肿块较大的肿瘤来说，诱导化疗可以缩小肿块从而减轻放疗毒性反应。来自加州大学的Gandara教授对该研究进行了评述：目前已有的证据表明早期放疗能给患者带来益处，但并不是说一定要在第1周期化疗开始的时候就给予，毕竟并不是所有的局限期SCLC都能耐受这种治疗方案。Park等进行的研究是对目前已有数据的重要补充，但该研究也存在着一些缺陷，比如该研究采用完全缓解率作为主要研究终点指标，这对一个Ⅲ期临床研究来说并不常见，因此对于OS的评估存在着统计能力不足的风险；另外该研究也未对延迟放疗时机可能产生的危害设立终点指标进行评价。

### 1.2 广泛期SCLC的治疗

#### 1.2.1 蒽环类药物治疗广泛期SCLC

蒽环类药物氨柔比星因其在SCLC的二线治疗中表现出的良好疗效而受到重视。Shipley等^[4]^进行了一项联合氨柔比星（30 mg/m^2^ d1- d3）、卡铂（卡铂浓度-时间曲线下面积=5，d1）、培非司亭（6 mg/m^2^ d4）一线治疗广泛期SCLC的临床Ⅱ期研究，主要观察终点是1年OS。该试验入组78例患者，7例完全缓解，47例部分缓解，12例疾病稳定；中位无进展生存期（progression-free survival, PFS）和OS分别是5.3个月和9.5个月，1年OS率为36%，显示出了氨柔比星联合卡铂用于一线治疗广泛期SCLC的有效性。

日本的JCOG9511试验^[1]^奠定了IP方案在SCLC治疗中的地位，但该方案常引起严重腹泻，而AP方案（氨柔比星+顺铂）在SCLC中有良好的应用前景且极少引起腹泻。一项来自日本的Ⅲ期临床研究（JCOG 0509）^[5]^比较了AP方案和IP方案治疗广泛期SCLC的疗效。该研究入组284例既往未接受化疗的广泛期SCLC患者，随机予以IP方案（伊立替康60 mg/m^2^ d1, d8, d15+顺铂60 mg/m^2^ d1，每4周为1个周期）或AP方案（氨柔比星40 mg/m^2^ d1-d3+顺铂60 mg/m^2^ d1，每3周为1个周期）治疗。主要研究终点是OS，次要终点是有效率、PFS、副反应、生活质量。在入组191例患者后，研究者发现发热性中性粒细胞减少症在AP组比预期多见，遂将氨柔比星剂量从40 mg/m^2^减小至35 mg/m^2^。中期分析显示，AP组中位生存期显著低于IP组（15.0个月*vs* 18.3个月，危险比=1.41），甚至超过了非劣性边缘，数据安全监察委员会建议提前发表结果。两组中位PFS和有效率无统计学差异，虽然IP组3级-4级腹泻发生率高于AP组（7.1% *vs* 1.4%），但4级中性粒细胞降低（22.5% *vs* 78.6%）、3级-4级发热性中性粒细胞减少症（10.7% *vs* 32.1%）的发生率均低于AP组，提示AP方案虽然腹泻少见，但骨髓抑制明显，与IP方案相比未见到其非劣性。

#### 1.2.2 化疗联合靶向药物治疗广泛期SCLC

SCLC患者血清胰岛素样生长因子（insulin like growth factor, IGF）浓度增加以及胰岛素样生长因子受体（IGF-receptor, IGFR）过度表达与SCLC细胞增殖密切相关，使得下调IGFR成为较有潜力的SCLC治疗方向。MK-0646是一种靶向IGFR的单克隆抗体，Ellis等^[6]^报道了一项MK-0646联合EP方案用于SCLC的Ⅰ期临床研究。12名受试者均接受顺铂25 g/m^2^联合依托泊苷100 mg/m^2^ d1-d3（每3周为1周期）化疗，其中一组患者（3例）同期接受5 mg/kg/w MK-0646靶向治疗，另一组患者（9例）接受10 mg/kg/w MK-0646靶向治疗。结果显示患者对10 mg/kg/w MK-0646仍然显示出良好的耐受性，在不良反应方面，除剂量依赖的高糖血症外，与单纯EP方案无统计学差异。但该研究为小样本Ⅰ期临床试验，且未探讨该方案对生存的影响，尚需进一步评价其应用价值。

#### 1.2.3 广泛期SCLC的胸部放射治疗

Wilson等^[7]^回顾性分析了胸部放疗对广泛期SCLC的疗效。该研究入组141例患者，发现化疗结束后接受胸部放疗与未接受胸部放疗患者PFS分别为7.8个月*vs* 5.4个月（*P*=0.003），中位OS为8.9个月*vs* 5.9个月（*P*=0.002）；而既往经4个-6个周期以铂类为基础化疗的患者接受胸部放疗与未经胸部放疗患者PFS分别为9.2个月*vs* 7.7个月（*P*=0.02），中位OS为10.2个月*vs* 7.7个月（*P*=0.02）。该研究认为胸部放疗在广泛期SCLC患者治疗中的价值值得进一步评估，正在进行的一项前瞻性随机对照Ⅲ期临床研究REST试验纳入以铂类为基础的化疗后出现肿瘤缩小的SCLC患者，并随机分入胸部放疗组和观察组，旨在评价广泛期SCLC患者化疗后胸部放疗的价值，结果值得期待。

## 2 SCLC的二线治疗

### 2.1 二线化疗

氨柔比星二线治疗难治性SCLC有较好的疗效，一项前瞻性研究^[8]^评价了氨柔比星联合卡铂治疗难治性SCLC的安全性和有效性。入组30例完成一线化疗后90 d内复发的SCLC患者，中位化疗周期数为4。结果显示总有效率34%，疾病控制率83%，中位PFS 3.5个月，中位OS 7.3个月。该研究提示氨柔比星联合卡铂治疗难治性SCLC有效。这是氨柔比星联合卡铂治疗难治性SCLC的首项前瞻性研究，氨柔比星是否能经得起大规模临床试验的考验还不得而知。

苯达莫司汀是一种烷化剂，既往研究^[9]^显示，苯达莫司汀联合卡铂一线治疗广泛期SCLC的有效率达73%，肿瘤进展时间为5.2个月，OS为8.3个月。美国一项开放、单组多中心Ⅱ期研究^[10]^对其二线治疗难治性SCLC进行了探索。研究共入组48例患者，接受苯达莫司汀120 mg/m^2^ d1, d2（每3周为1个周期）化疗。结果显示33例可评价疗效，其中1例完全缓解，9例部分缓解，13例疾病稳定（疾病控制率为48%），10例疾病进展；中位肿瘤进展时间为3.37个月。至数据分析时，13例患者存活，中位OS 4.77个月。3度-4度不良事件包括乏力（18.8%）、气促（14.5%）、无中性粒细胞降低的感染（12.5%）、贫血（8.3%）、中性粒细胞降低（8.3%）和腹泻（8.3%），提示单药苯达莫司汀二线或三线治疗SCLC有效且耐受性良好。

此外，DNA修复基因甲基鸟嘌呤DNA甲基转移酶（methylguanine-DNA methyltransferase, MGMT）是肿瘤耐受烷化剂药物的主要原因，靶向MGMT修复机制克服肿瘤组织耐药正成为肿瘤治疗的一个新途径。美国研究者Drilon等^[11]^报告了替莫唑胺5 d方案在SCLC二线治疗的研究。替莫唑胺的剂量为200 mg/m^2^（d1-d5，28 d为1个周期）。结果显示总有效率为12%，中位PFS和OS分别为1.3个月和7.9个月。该试验证实了MGMT阴性患者较阳性患者对替莫唑胺更为敏感。

### 2.2 二线靶向治疗

阿柏西普是一种重组人融合蛋白，与循环血管内皮生长因子紧密结合，使其不能与细胞表面受体相互作用。Allen等^[12]^共报告了98例可评估的铂类耐药、广泛期或局限期SCLC患者，随机给予其单纯拓扑替康（4 mg/m^2^ d1, d8, d15）或联合阿柏西普（6 mg/kg d1）治疗。拓扑替康给药方案一般为每个周期连续5 d每日给药，但研究者选择了每周1次给药方案以减轻毒性反应。联合阿柏西普治疗组3个月PFS明显高于单纯拓扑替康治疗组，但联合治疗组和单纯拓扑替康组的OS无统计学差异（4.6个月*vs* 3.9个月，*P*=0.25）。该研究参与者Gandara指出“抗血管生成药物可能会在实体瘤疗效评价标准未显示缓解的情况下已经显示生物学活性”。抗血管生成治疗的作用并非使肿瘤迅速缩小，而是使之长期稳定，故评价细胞毒药物疗效的RECIST标准不一定适用。

帕唑帕尼是血管内皮生长因子受体1-3、血小板衍生生长因子受体和细胞因子受体等受体的酪氨酸激酶抑制剂。一项来自美国曼撤斯特总医院癌症中心的临床Ⅱ期研究^[13]^探讨了帕唑帕尼作为SCLC二线药物的疗效。入组27例二线治疗后出现疾病进展的患者，接受每日800 mg的帕唑帕尼单药治疗，主要终点为第8周的PFS。结果显示第8周PFS率高达52%，中位PFS也超过历史记录（< 2个月）达到14.1周。从这个结果来看，帕唑帕尼是一种很有潜力的药物，当然这仅仅是Ⅱ期临床结果，其临床价值有待进一步评估。

化疗药物和基因治疗缺少肿瘤靶向性以致疗效有所降低，因此提高治疗药物和载体的肿瘤靶向性是提高疗效的关键。天冬酰胺-甘氨酸-精氨酸-人肿瘤坏死因子（NGR-hTNF）能选择性破坏肿瘤血管，与多柔比星联合能提高瘤内多柔比星浓度。意大利一项Ⅱ期研究^[14]^探索了NGR-hTNF联合多柔比星治疗复发性SCLC的疗效。研究共纳入28例一线或一线以上含铂化疗方案失败的SCLC患者。结果显示，中位PFS为3.2个月，1年OS率为34%，疾病控制率达55%；既往接受二线及以上治疗的患者，疾病控制率达75%，中位PFS为5.1个月，1年OS率为44%；铂类耐药或敏感的患者，疾病控制率分别为50%和58%（*P*=0.72），中位PFS分别为2.7个月和4.1个月（*P*=0.07），1年OS率分别为27%和42%（*P*=0.65）。该研究提示联合方案对铂类敏感和耐药的SCLC均有效。

## 3 SCLC的预后因素

目前尚缺乏SCLC有效的预后因子。韩国国家癌症中心开展了一项研究^[15]^，对139例IP方案化疗后的广泛期SCLC患者进行了全基因组测序，探讨OS和基因之间的关系，寻找可能的预后基因。结果发现rs16950650 CT、rs7186128 AG或GG、rs17574269 AG、rs8020368 CC、rs4655567 CC、rs2166219 TT和rs2018683 TT等7个单核苷酸多态性与OS降低相关，其中rs4655567、rs8020368、rs2018683和rs17574269与复发、转移密切相关，但这一研究结果与临床应用仍有距离，我们在此新兴领域还需进一步探索。

循环肿瘤细胞作为一种生物标志物被认为是恶性肿瘤出现复发和远处转移的重要原因，具有用于SCLC的疗效预测和预后判断的潜力，有助于进一步研究SCLC的起源、疾病复发和转移机制。Ranganathan等^[16]^进行了一项前瞻性研究对新诊断的21例SCLC患者进行循环肿瘤细胞计数，并将其与疾病分期、转移灶数目、对治疗敏感性、疾病进展时间作相关性分析。结果表明高循环肿瘤细胞数量与疾病分期晚（局限期*vs*广泛期：1 *vs* 80.5）、转移灶数目多（1个-2个转移灶*vs* 3个以上转移灶：71 *vs* 2, 668）相关，且对放化疗敏感的患者循环肿瘤细胞数目会相应降低。该研究还在进行中，循环肿瘤细胞与肿瘤进展时间的相关性数据期待在下一届ASCO会议上公布。

## 4 基础研究

人第10号染色体的磷酸酶及张力蛋白同源的基因缺失（phosphatase and tensin homolog deleted on chromosome 10, PTEN）与磷脂酰肌醇3-激酶（phosphoinositide-3-kinase, PI3K）的缺陷在SCLC中十分常见，因此阻断PTEN/PI3K/蛋白激酶（PTEN/PI3K/AKT）通路是SCLC治疗的可行途径。MK-2206是一种强效、高特异性及非ATP竞争结合的AKT变构抑制剂。Holland等^[17]^报告了一项拓扑替康或MK-2206或联合用于SCLC细胞株的实验。细胞株选择H526-PTEN/PI3K野生型、H69-PTEN野生型/PI3K突变型和H2196-PTEN突变型/PI3K野生型。H526细胞株拓扑替康治疗有效，MK-2206治疗无效；相反，H69细胞株对MK-2206敏感，对拓扑替康耐药；而H2196细胞株对两种药物均敏感。该研究表明MK-2206对PTEN或PI3K突变的细胞株有效，与拓扑替康联用于野生型或突变型细胞株均能提高其敏感性。基于这些数据，一项比较拓扑替康联合MK-2206与拓扑替康单药治疗复发性SCLC的美国西南肿瘤学组（Southwest Oncology Group, SWOG）的临床试验正在筹备中。

我们知道SCLC细胞或肿瘤中有DNA修复蛋白聚合酶1（Poly [ADP-ribose] polymerase 1, PARP1）表达水平上升。Byers等^[18]^报道了PARP1抑制剂（奥拉伯尼或AG014699）治疗SCLC的一项临床前期试验。结果显示SCLC细胞株对两种药物均十分敏感，而奥拉伯尼联合拓扑替康或依托泊苷较任一单药更好地降低了肿瘤细胞株的存活率（*P* < 0.03）。该研究表明DNA修复酶抑制剂作为SCLC的新药有广阔的前景。

## 5 结语

综上所述，4个周期EP方案化疗同步加速超分割胸部放疗仍是局限期SCLC标准治疗方案，但最佳的胸部放疗时机仍未明确。对于广泛期SCLC，一线使用氨柔比星骨髓抑制明显，与IP方案相比未显示出非劣性；胸部放疗的地位需要进一步评估；单克隆抗体MK-0646在广泛期SCLC的治疗中有一定的价值。在SCLC的二线治疗中，氨柔比星、苯达莫司汀以及替莫唑胺显示出了较好的有效性和安全性；肿瘤血管生成抑制药物阿柏西普、帕唑帕尼以及载体药物NGR-hTNF在SCLC靶向治疗方面具有潜在的应用前景。SCLC患者循环肿瘤细胞水平有可能是重要预后指标，而MK-2206、PARP1抑制剂有可能成为SCLC未来靶向治疗的重要药物。
